# Regarding mentorship

**DOI:** 10.1038/s41467-020-20618-x

**Published:** 2020-12-21

## Abstract

The publication of a paper on mentorship, now retracted, led us to reflect on our editorial processes and strengthened our determination in supporting diversity, equity and inclusion in research.

On November 17^th^, 2020 we published an Article entitled “The association between early career informal mentorship in academic collaborations and junior author performance” which immediately attracted significant criticisms from readers. Mainly, these were directed to claims of a negative impact of both female mentors and mentees and suggestions that opposite-gender mentorship may help to elevate the status of women in science. Following a journal investigation, the paper has now been retracted by the authors as explained below.

We selected the paper for publication because we believe that mentorship in academia is an important and understudied area of research. Quantifying institutional biases in science publication, citation and “impact” is a necessary first step towards recognising and addressing them. This paper analysed a large dataset to approach the question of collaboration and co-authorship among junior and senior researchers in an attempt to study informal mentorship, and we considered this to be of interest to the community.

The criticisms from readers revolved around the validity of the conclusions in light of the available data, assumptions made and methodology used. In particular, readers criticised the use of co-authorship as a measure of mentorship, and citations as a measure of success of the mentoring relationship. Some of these concerns were also raised during the first round of peer review, which involved four reviewers with expertise ranging from science of science, network analysis and mentoring relationships. In light of these concerns, a number of changes, including the addition of a survey that was aimed at elucidating the type of mentorship between pairs of junior and senior co-authors, were added to the revised version by the authors and the manuscript was peer reviewed again. Upon publication, it became clear that the concerns had not been sufficiently addressed, and we started an investigation. We alerted readers with an Editor’s Note published on November 19^th^.

We followed our established editorial processes, which involved recruiting three additional independent experts to evaluate the validity of the approaches and the soundness of the interpretation. They supported previous criticisms and identified further shortcomings in relation to the use of co-authorship as a measure of informal mentorship. They also noted that the operationalisation of mentorship quality, based on the number of citations and network centrality of mentors, was not validated.

According to these criticisms, any conclusions that might be drawn on biases in citations in the context of co-authorship cannot be extended to informal mentorship. As such, the paper’s conclusions in their current form do not stand, and the authors have retracted the paper.

During the investigation, we also received further communications from readers highlighting issues with the paper and are grateful to all the researchers who have contacted us and who have invested their time in reviewing the work.

Simply being uncomfortable with the conclusions of a published paper, would and should not lead to retraction on this basis alone. If the research question is important, and the conclusions sound and valid, however controversial, there can be merit in sharing them with the research community so that a debate can ensue and a range of possible solutions be proposed. In this case, the conclusions turned out not to be supported, and we apologise to the research community for any unintended harm derived from the publication of this paper.

As part of our investigation, we also reviewed our editorial practices and policies and, in the past few weeks, have developed additional internal guidelines, and updated information for authors on how we approach this type of paper. As part of these guidelines, we recognise that it is essential to ensure that such studies are considered from multiple perspectives including from groups concerned by the findings. We believe that this will help us ensure that the review process takes into account the dimension of potential harm, and that claims are moderated by a consideration of limitations when conclusions have potential policy implications. We will keep developing our guidelines for manuscripts with sensitive research in the social and behavioural sciences, and in areas with significant societal and public policy impact.

This experience has reinforced our commitment to equity and inclusion in research. We have been working to strengthen our ongoing efforts to reach out to a diverse pool of reviewers and commissioned authors. In collaboration with Sense about Science, we launched as a pilot a peer review programme for early career researchers, consisting of a webinar and a hands-on phase which we plan to extend next year. We will share the results of these efforts in editorials in 2021.

As part of the longstanding commitment to mentorship that characterises the Nature journals, we intend to highlight, recognize and support mentorship by women academics. We are actively discussing within the editorial team what further initiatives we can launch or support and we will finalise our plans and commitment early next year.

